# Strong Dependence between Tryptophan-Related Fluorescence of Urine and Malignant Melanoma

**DOI:** 10.3390/ijms22041884

**Published:** 2021-02-13

**Authors:** Anna Birková, Marcela Valko-Rokytovská, Beáta Hubková, Marianna Zábavníková, Mária Mareková

**Affiliations:** 1 Department of Medical and Clinical Biochemistry, Faculty of Medicine, Pavol Jozef Šafárik University in Košice, Tr. SNP 1, 040 11 Košice, Slovakia; anna.birkova@upjs.sk (A.B.); maria.marekova@upjs.sk (M.M.); 2 Department of Chemistry, Biochemistry and Biophysics, University of Veterinary Medicine and Pharmacy in Košice, Komenského 73, 041 81 Košice, Slovakia; 3 KOREKTCHIR s.r.o., Zborovská 7, 040 01 Košice, Slovakia; marianna.zabavnikova@gmail.com

**Keywords:** malignant melanoma, urine, autofluorescence

## Abstract

Urine autofluorescence at 295 nm is significantly higher in patients with malignant melanoma at each clinical stage compared to the healthy group. The largest difference is in the early-stages and without metastases. With increasing stage, the autofluorescence at 295 nm decreases. There is also a significant negative correlation between autofluorescence and Clark classification. Based on our results, it is assumed that the way malignant melanoma grows also affects urinary autofluorescence.

## 1. Introduction

Human urine is a complex biological fluid containing a variety of both endogenous and exogenous chemical compounds excreted by the body. The optical properties of the biological system obtained by fluorescence analysis reflect some of the physicochemical properties of the metabolites. Urine is a multicomponent mixture of different fluorophores and nonfluorescent metabolites [1,2,3,4].

In modern medicine, the use of urine as one of the biological fluids is very widespread because it does not require invasive sampling. Under physiological conditions, the range of urine output is 800 to 2000 mL per day, providing a sufficient amount of urine for sampling. There are more than 1700 metabolites identified and quantified in the human urine, and other almost 3000 expected or identified, but not quantified metabolites, listed in the Urine Metabolome Database with their structures and links to their known health and disease associations [4]. Urine analysis is advantageous over other biological matrices because the metabolites present, mostly those representing the final breakdown products of foods and beverages, technological additives, drugs, environmental contaminants, and even endogenous waste metabolites and bacterial byproducts, are more stable in the urine. Urine requires minimal pretreatment of the sample and reflect the physiological/pathological state of the biological system [5].

In recent years, progress has been made in the use of fluorescence spectroscopic techniques for the diagnosis of selected oncological diseases, such as ovarian cancer [2,6,7], pancreatic cancer [8], breast cancer [9,10], bladder cancer [11].

The incidence of skin cancer—melanoma—is increasing worldwide, and it represents 3% of all skin cancers but 65% of skin cancer deaths [12]. Melanoma is currently the fifth and sixth most common solid malignancy diagnosed both in men and women [13]. Malignant melanoma is a neoplasm derived from specialized melanin-producing cells called melanocytes or cells that develop from melanocytes. Melanoma spreads through the lymphatic system and blood and can therefore metastasize to any organ in the body. For this reason, early detection is very important. Malignant melanoma is a tumor with a high production of melanin. Precursors of melanin and their metabolites play an important role in melanogenesis, but in a broader sense, melanogenesis also refers to the process of melanosome formation and transfer to the keratinocytes. While melanins are polymers, the whole process of their synthesis, storage and transport takes place in the organelles called melanosomes. Before generating a sufficient amount of melanins to be seen by light microscopy, the melanosomes are known as pre-melanosomes.

Melanins are synthesized from the precursor amino acid L-tyrosine. It is hydroxylated by tyrosinase in the presence of dioxygen to l-dihydroxyphenylalanine (L-DOPA) and consequently to dopaquinone (melanogenic pathway, Raper–Mason pathway). In the following step, dopaquinone is metabolized to dopachrome by rapid cyclization. Dopachrome can be decarboxylated to 5,6-dihydroxyindole (DHI) or tautomerized to 5,6-dihydroxyindole-2-carboxylic acid (DHICA). Intermediates DHI and DHICA are oxidized to form eumelanin, with brown and black subtype [14].

Red-orange pheomelanins are synthesized after conjugation with cysteine and the subsequent oxidation of the intermediate. Neuromelanin, which has an approved role in aging and in the development of Parkinson’s disease, can be formed both from eumelanin and from pheomelanin intermediates in dopamine and norepinephrine neurons [15].

Some studies have shown that, in addition to tyrosine, tryptophan and some of its metabolites are also involved in melanin biosynthesis [16,17,18]. Serotonin and its metabolite 5-hydroxyindole-3-acetic acid (5-HIAA) occurring in urine act as specific tumor markers, and increased production of 5-HIAA have been documented in human epidermal keratinocytes and melanoma cells [17].

Many of these metabolites have native fluorescent properties and are part of biological samples such as tissue, blood, and urine. Metabolism of cancerous tissues differs from healthy ones, which also affects the composition of natural fluorophores in body biofluids.

The use of urinary fluorescence analysis offers the possibility to detect urinary metabolites potentially associated with the neoplastic process, which could provide a new direction in the current search for predictive and prognostic markers [2,11,19,20]. In our previous studies, we have confirmed the role of fluorescence spectroscopy as a useful diagnostic tool with high-efficiency in ovarian cancer [7] as well as the feasibility of synovial fluid fluorescence fingerprinting to identify disease-specific profiles of synovial fluid metabolites [21].

In this study, we focused on the analysis of changes in urine autofluorescence from patients with malignant melanoma in comparison to healthy subjects as a tool of non-invasive melanoma detection.

## 2. Results

### 2.1. Fluorescence Measurements in Control and Malignant Melanoma Group

**Figure 1:**
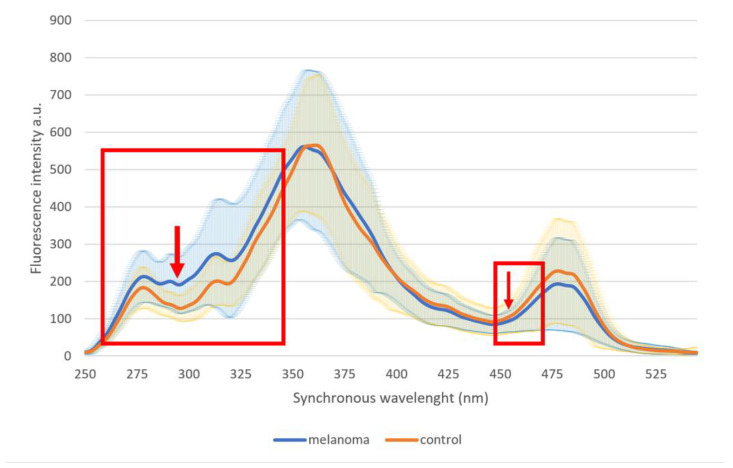
Average fluorescence profiles of urine in the control group and whole malignant melanoma group. Rectangles indicate wavelength areas with significant differences; arrows indicate wavelengths with the largest statistical difference.

**Figure 2:**
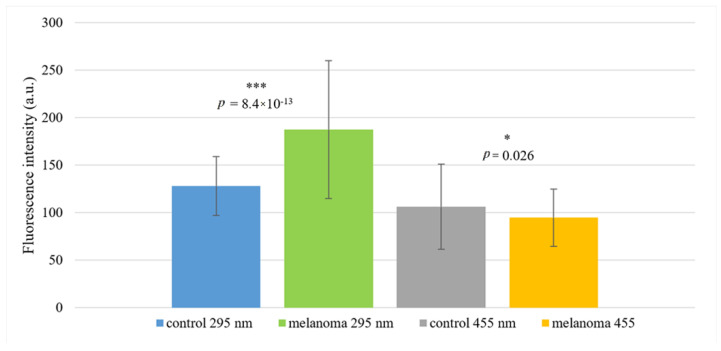
Fluorescence intensities of the control and malignant melanoma groups at 295 nm and 455 nm. Statistically significant differences are related to the control group. Values are expressed as mean ± SD. * indicates *p* < 0.05 and *** indicates *p* < 0.001.

Statistically significant differences were found in two wide spectral regions at region 254–348 nm (with the most significant difference at 289–302 nm, *p* = 8.4 × 10^−13^) and at region 451–470 nm (with the most significant difference at 455–458 nm, *p* = 0.025) (Figure 1, Table 1, Figure 2).

### 2.2. Confrontation of Fluorescence Analysis and Histological Findings in Malignant Melanoma Group

In the subsequent analysis, we focused mainly on the area around the wavelength 295 nm, as in this region was found the most significant difference between the control and melanoma group (*p* = 8.4 × 10^−13^).

**Figure 3:**
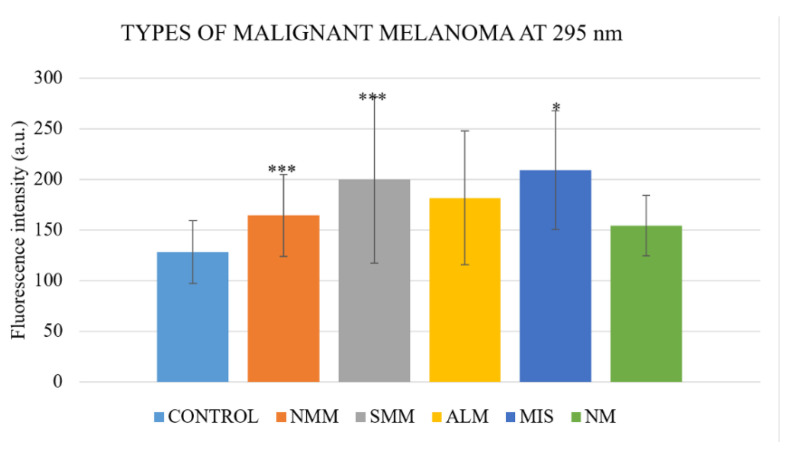
Fluorescence intensity in different types of malignant melanoma. NMM—nodular type malignant melanoma, SMM—superficial spreading-type melanoma, ALM—acral lentiginous malignant melanoma and lentigo maligna group, MIS—melanoma in situ, NM—nevoid melanoma. Statistically significant differences are related to the control group. Values are expressed as mean ± SD. * indicates *p* < 0.05 and *** indicates *p* < 0.001.

When analyzing according to the type of the malignant melanoma, there were strong significant differences between control group and nodular-type malignant melanoma group (*N* = 26; *p* = 1.64 × 10^−6^), superficial-spreading-type group (*N* = 54; *p* = 5.63 × 10^−8^), and weak difference in melanoma in situ group (*N* = 6; *p* = 0.01), but not between control group and acral lentiginous malignant melanoma and lentigo maligna group (*N* = 7; *p* = 0.077) or nevoid melanoma (*N* = 3; *p* = 0.1; Table 2, Figure 3).

**Figure 4:**
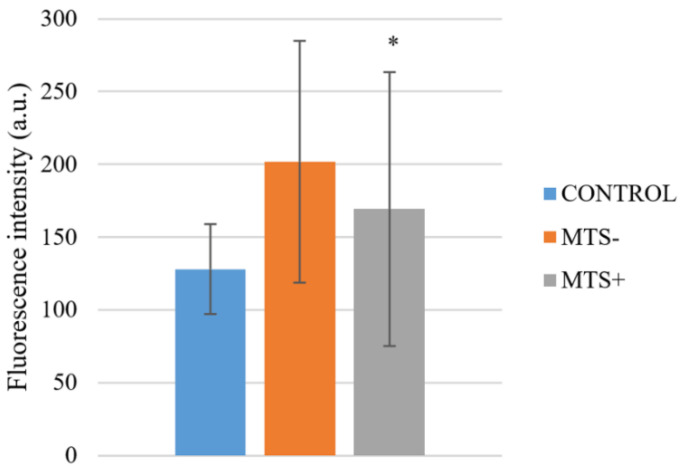
Fluorescence intensity in patients with malignant melanoma with (MTS+) or without metastases (MTS-) compared to the control. Statistically significant difference is between MTS+ and MTS- group. Values are expressed as mean ± SD. * indicates *p* < 0.05.

Within the melanoma group, there were found significant differences between fluorescence intensity in nodular-type malignant melanoma group and superficial-spreading-type melanoma (*p* = 0.037), and nodular-type malignant melanoma group and melanoma in situ (*p* = 0.016). There was not a significant difference (*p* = 0.52) in fluorescence intensity of melanoma group at 295 nm between those who had in the histological finding of ulceration or not, nor those with positive or negative HMB-45 and MelanA marker. MelanA and HMB-45 are commonly determined differentiation antigens in malignant melanoma, and their loss is relatively common, especially in progressive disease with metastatic lesions. When evaluating the effect of metastases on fluorescence analysis, it shows a significant difference (*p* = 0.013) in fluorescence intensity at 295 nm between those with (*N* = 34, 169.2 ± 94.1) or without (*N* = 71, 201.8 ± 83.1) metastases (Figure 4).

**Figure 5:**
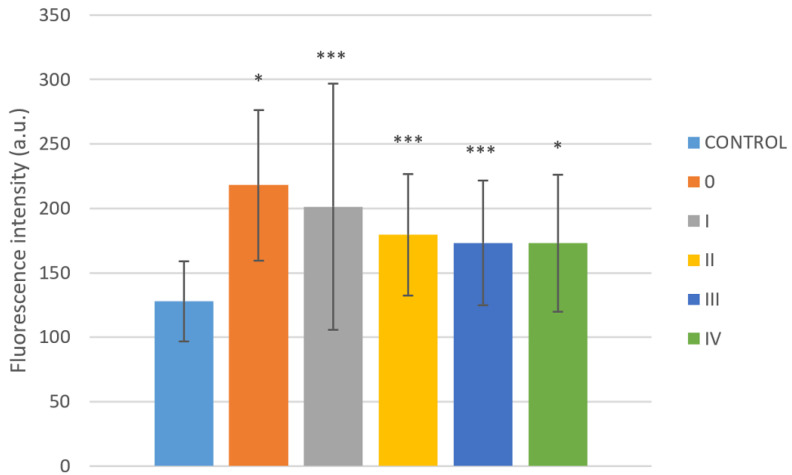
Fluorescence intensity in various clinical stages. Statistically significant difference is related to the control group. Values are expressed as mean ± SD. * indicates *p* < 0.05 and *** indicates *p* < 0.001.

Analysis of clinical stage relation to fluorescence shows significant difference between fluorescence at 295 nm of the control group and particular stage (0-IV), but with different significance (0–IV: *p* = 0.013, 0.000014, 0.000033, 0.0002 and 0.026, respectively, Figure 5). The highest fluorescence intensity was at stage 0 (*N* = 6, 218.1 ± 58.4) and decreases with increasing clinical stage (I: *N*=42, 201.2 ± 95.5, II: *N*=24, 179.4 ± 47.2, III: *N*=23, 173.2 ± 48.3, IV: *N*= 10, 173.0 ± 53.4).

**Figure 6:**
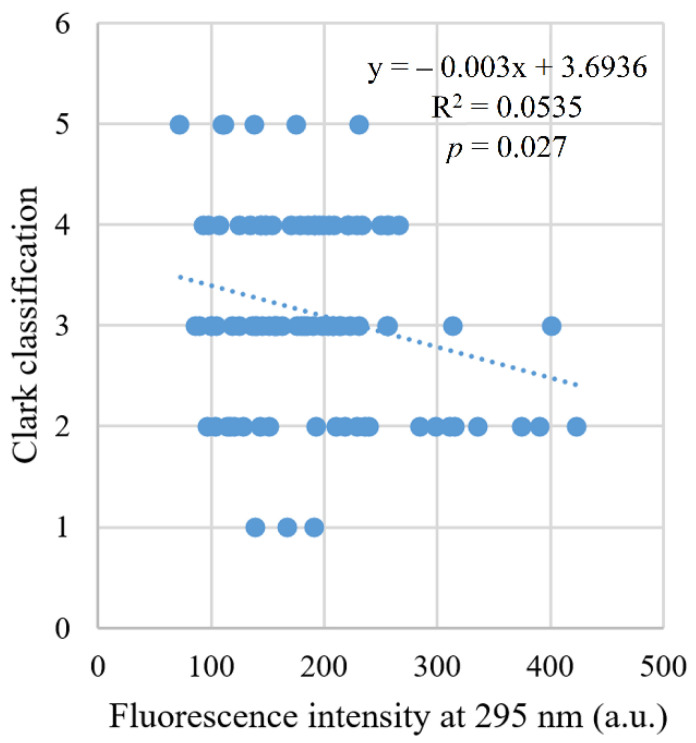
Correlation between fluorescence intensity and Clark scale.

**Figure 7:**
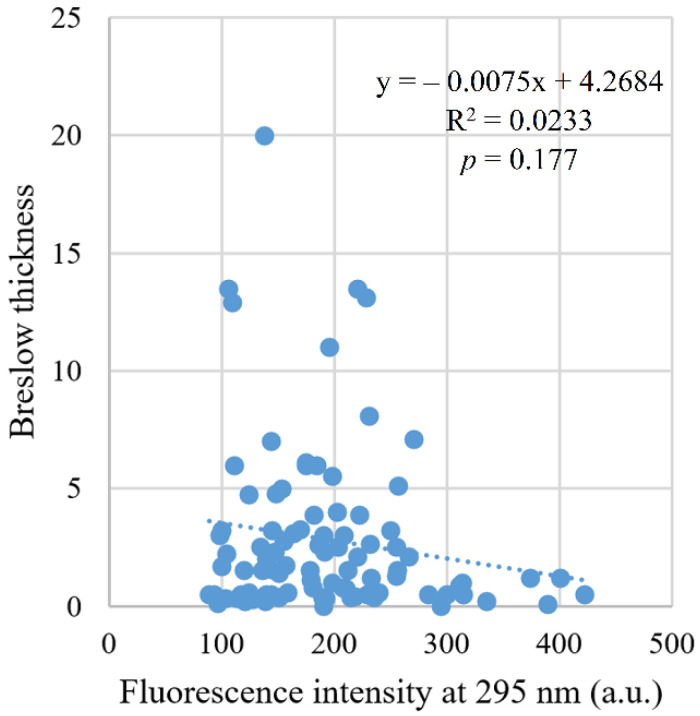
Correlation between fluorescence intensity and Breslow thickness.

A significant negative correlation was found (*r* = −0.20, *p* = 0.041) between fluorescence intensity and clinical stage. There was also a significant negative correlation of fluorescence intensity (*r* = −0.23, *p* = 0.027) with Clark level of invasion (Figure 6), but not with Breslow thickness (*r* = −0.14, *p* = 0.177, Figure 7).

## 3. Discussion

Malignant melanoma is a type of cancer with high metastatic potential, and early detection is crucial for the later assessment of prognosis and survival of patients. In our study, we have found that the urine of patients with malignant melanoma has higher fluorescence at a wide range of wavelengths, with the most significant difference at 295 nm, and the early-stages have higher fluorescence than the later ones. There exists very little information on the use of native fluorescence in malignant melanoma. In vivo diagnostics of skin, lesions have been published only a few times in the past. Chwirot et al. [22] tested the sensitivity of fluorescence detection of melanoma compared to other pigmented lesions. The tested wavelengths showed at ex/em 366/475 nm 82.7% sensitivity and 59.9% specificity. The authors concluded that the origin of the observed autofluorescence is not fully understood, and a plausible hypothesis may be that the spatial distributions of the autofluorescence observed for the skin (suggested fluorophores could be collagen in combination with elastin and desmosine or keratin) surrounding the pigmented lesions may result from responses of the host cells reacting to the presence of the lesions. In 2006, Borisova reported the high distinguishing potential of a fluorescence signal measured from skin lesions in vivo by a fiber-optic spectrophotometer. The most significant difference was found at similar wavelengths ex/em 337/480–490 nm, and the author reported a possible distinction between benign nevi and malignant melanoma [23]. The lower fluorescence of the reported area was interpreted by the author as a consequence of the hypervascularization of malignant neoplasia and thus the hemoglobin quenching effect. Lower fluorescence around 450 nm in melanoma patient’s urine was also detected in this study, but we can exclude the effect of hemoglobin, as the urine samples were checked on the presence of the blood using strip tests and were blood negative. On the other hand, in vivo diagnostics of pigmented lesions can also be dangerous, especially upon excitation of the light in the UV area [24,25]. It appears to be a safer out-of-body autofluorescence examination or measurement of the biological fluids. Recently, was published a paper in which the authors focused on the measurement of analysis of the urine in malignant melanoma patients after removing NADH fluorescence by glutathione reductase derivation, and some results are also related to underived urine autofluorescence [26]. They focused on the range of 300–500 nm and described significantly lower emission at 460 nm in melanoma patients compared to the control group. The lower fluorescence emission at 460 nm in underived urines of melanoma patients correlates with our lower fluorescence at 450 nm, which differs slightly from the control group. An additional common finding of the compared study is that there was no difference in urine fluorescence in MelanA positive or negative patients, but when comparing positive or negative ulceration, there was a difference, which is not in correlation with the result of our study with *p* = 0.76. The divergence in this result probably stems from the fact that the study of Špaková et al. recruited 56 people (46 melanoma patients and 10 controls), while our study included more than 220 people (105 melanoma patients and 119 controls).

In this study, we focused more on the wavelength at 295 nm as there was a very significant difference between the healthy control group and the melanoma group (*p* = 8.4 × 10^−13^). Fluorescence is higher in all clinical stages of malignant melanoma, but the difference at early stages, even in the in situ stages of melanoma, with a Breslow thickness equal to zero, is very interesting. The clinical stage correlates slightly negatively with fluorescence, and this is also in agreement with a larger difference compared to the control group and patients without metastases than with metastases. The Breslow thickness is not related, but the Clark invasion is related to fluorescence, again with a negative correlation. Based on results, the type of malignant melanoma also plays a role in influencing fluorescence, but regardless of the positivity or negativity of the markers Melan A and HMB-45. The fluorescence area around 295 nm is typical for the fluorescence of proteins and is close to the fluorescence of amino acids tryptophan and phenylalanine and their metabolites and derivatives [27,28]. Malignant melanoma is associated with many molecules that could hypothetically fluoresce in this area. Recently, Belter et al. reported a list of potential biochemical serum markers that could be useful in diagnosing or assessing the prognosis of melanoma cancer patients. Among these markers are large molecules such as many proteins with functions such as enzymes, antigens, growth factors, inflammatory markers, e.g., lactate dehydrogenase, tyrosinase, cyclooxygenase 2, many matrix metalloproteinases, indoleamine-2,3-dioxygenase, tissue inhibitor of metalloproteinase, vascular endothelial growth factor, osteopontin, C-reactive protein, etc. [29], but also small molecules that are precursors of eumelanin and pheomelanin, such as 5-S cysteinyldopa, 6-hydroxy-5-methoxyindole-2-carboxylic acid, 5,6-dihydroxyindole, 5,6-dihydroxyindole-2-carboxylic acid [30,31]. Other melanoma-related derivatives of amino acids phenylalanine and tryptophan are also described, such as vanilmandelic acid, homovanilic acid, 5-hydroxyindole-3-acetic acid and indoxyl sulfate [18]. Many of these mentioned small molecules fluoresce naturally and are also present in urine. The amino acids phenylalanine and tryptophan and their metabolites have similar fluorescent properties in the characteristic fluorescence zone of 250–350 nm. The major fluorophore in this fluorescent area is tryptophan, and its derivative, indoxyl sulfate, is also present. Tryptophan is the most abundant component among the three fluorescent amino acid components of proteins. The contribution of phenylalanine to the intrinsic fluorescence of the protein is negligible due to its low absorption and very low quantum yield. Tyrosine has a quantum yield similar to tryptophan, but the tryptophan indole group is considered to be the dominant source of UV absorption at 280 nm and emission at 350 nm in proteins [32]. It is also possible that some proteins can contribute to increased fluorescence at 295 nm. The already mentioned amino acids and many of their metabolites with fluorescent characteristics are naturally present in the urine, and their presence is associated with several different diseases [3,7,20,33]. Matrix metalloproteinases are present in the urine in some cancer types, and their combination is cancer-specific [34]; some are specific for melanoma [35]. Based on our ongoing research, we believe that the increased urine fluorescence at 295 nm is related to the increased tryptophan concentration, which can be derived from free tryptophan as well as from tryptophan-rich protein degradation. This unpublished research pointed to a significant positive correlation between urinary tryptophan concentration and fluorescence at 295 nm (*r* = 0.24, *p* = 0.041; data not published).

## 4. Materials and Methods

### 4.1. Composition of the Study Group

The study group consisted of 105 patients with malignant melanoma with clinical stage 0 to IV; average age 56 ± 15.47 years; 52 men (49.5%) and 53 women (50.5%). The Control group consisted of 119 healthy controls (average age 40.4 ± 11 years; 75 men (63%) and 44 women (27%). Patients were recruited during hospitalization at the Department of Plastic and Reconstructive Surgery UPJŠ LF in Košice. The diagnosis of malignant melanoma was confirmed histologically, and the melanoma staging was based on the eighth edition of the American Joint Committee on Cancer (AJCC) staging system [36] that uses the following key information for assigning Tumor-Node-Metastasis (TNM) classifications: Breslow thickness of the tumor, ulceration presence, depth invasion by Clark scale and the presence of nodal or distant metastases. The healthy control group was chosen by random assignment, with the following criteria: the absence of any oncological disease, negative oncological family anamnesis and any serious disease (Table 3).

The study participants were not following any special diet and were asked not to take vitamin supplements prior to urine collection. Other medicaments and drugs prescribed by the general practitioner were retained. The use of drugs by probands was sporadic and statistically unevaluable, so we neglected the effect of the drugs in the present study.

### 4.2. Urine Samples

Urine samples were obtained from patients with malignant melanoma immediately following admission to the hospital. Participants of healthy controls were given a morning appointment and asked to fast at least 8 h before the sample collection. Samples were taken under standard conditions as first morning urines. Leukocytes, nitrite, pH, specific gravity, protein, glucose, ketones, urobilinogen, bilirubin, blood were evaluated semiquantitative using the 10 parameter urine strip test Dekaphan Leuco (Erba Lachema, Brno, Czech Republic). The average values of the pH and the specific gravity in the healthy control group were 5.76 ± 0.62, and 1021.5 ± 8.2, respectively. Ketones were detected in 6 members of the healthy control group, in an amount of 1.5 mmol/L. Leukocytes, nitrite, protein, glucose, urobilinogen, bilirubin and blood were negative. The result of the listed urine parameters in the malignant melanoma patients’ group was not significantly different. The average pH and specific gravity values were 5.82 ± 0.50 and 1015.5 ± 10.5, respectively. Subsequently, the samples were stored at −27 °C. After thawing and centrifugation at 5000 rpm for 10 min at laboratory temperature (Centrifuge Boeco S8, Boeco Germany, Hamburg, Germany), urine samples were analyzed.

### 4.3. Instrumentation and Statistical Analysis

The autofluorescence of urine samples was measured at room temperature, using a Luminescence Spectrophotometer PerkinElmer LS55 (PerkinElmer, Waltham, Massachusetts, USA) in 1 cm quartz cuvettes (Helmut Fischer, Sindelfingen, Germany). From every sample, we measured 12 synchronous spectra with Δλ 30 nm in the range 250–550, step 0.5 nm, excitation/emission slits 5/5 nm, and a scan speed of 1200 nm/min. The measurements were performed, and urine fluorescence profiles were constructed as previously published [2].

Statistical analysis was performed with SPSS Statistics software version 22 (IBM, Armonk, New York, USA). The F-test was used to assess whether the standard deviations within the groups differ from each other. A Student’s unpaired *t-*test was performed to determine the differences in fluorescence intensity within the control group and various descriptive parameters in malignant melanoma patients. A Student’s unpaired *t-*test was also used to analyze the differences in fluorescence intensities between the types of malignant melanoma compared to the control group. Pearson’s correlation analysis was performed to demonstrate the strength and direction of the linear relationship between fluorescence intensity and clinical stage, Clark scale or Breslow thickness. Statistical significance was assigned for *p*-value < 0.05. Values of fluorescence intensity were expressed as mean ± SD.

## 5. Conclusions

Fluorescence spectrometry has become a standard tool in many areas of research and is a universal alternative technique for studies using radioactive labels. A large number of fluorescence techniques and methods have been developed to study biological processes. Autofluorescence of biological systems, such as urine, has facilitated the development and review of several approaches without the necessary labeling. Significantly higher autofluorescence of the urine at 295 nm in melanoma patients may be useful background for further studies revealing metabolic changes in malignant melanoma, but may also serve as a potential diagnostic marker, as the early-stages show even more pronounced changes in tryptophan-related fluorescence intensity than the later ones.

## Figures and Tables

**Table 1:** Comparison of fluorescence intensity at various wavelengths through whole measured spectrum (in 25 nm steps) in healthy and control group. For evaluation of differences, Student’s *t*-test was used. Values of fluorescence intensity are expressed as mean ± SD.

λ (nm)	Group		*p*-Value of Student’s *t*-Test
	Control	Malignant Melanoma	
250	9.8 ± 2.6	9.6 ± 5.1	0.74
275	171.4 ± 51.2	199.7 ± 68.8	0.00069
300	134.8 ± 40.7	204.4 ± 85.7	3.5 × 10^−12^
325	228.1 ± 78.9	273.1 ± 146.2	0.0056
350	492.9 ± 128.5	527.5 ± 175.9	0.098
375	417.4 ± 169.2	440.8 ± 201.3	0.35
400	216.1 ± 95.7	215.0 ± 68.8	0.92
425	132.1 ± 53.0	121.8 ± 42.2	0.11
450	94.7 ± 37.8	86.7 ± 24.7	0.059
475	220.6 ± 134.5	187.4 ± 117.1	0.052
500	85.0 ± 64.4	74.5 ± 41.6	0.14
525	14.9 ± 8.7	17.0 ± 12.3	0.15
550	9.4 ± 15.3	8.2 ± 4.2	0.42

**Table 2:** Statistical analysis of types of malignant melanoma compared to the control group. For evaluation of differences, Student’s *t-*test was used. Values of fluorescence intensity are expressed as mean ± SD.

	Control	Nodular Type	Superficial Spreading Type	Acral Lentiginous Melanoma + Lentigo Maligna	Melanoma In Situ	Nevoid Type
*N*	119	26	54	7	6	3
Fluorescence intensity Mean ± SD	127.9 ± 31.0	164.3 ± 40.6	199.5 ± 82.1	181.5 ± 65.9	208.9 ± 58.7	154.3 ± 29.8
*t-*test		1 × 10^−5^	5.6 × 10^−8^	0.077	0.011	0.097

**Table 3:** Descriptive statistics of the study group.

	N	Age in Years		
		Minimum	Maximum	Mean
Healthy Control	119	22	65	40.4 ± 11
Malignant Melanoma	105	17	87	56.0 ± 15.5
Clinical Stage 0	6	17	64	45.2 ± 20.2
Clinical Stage I	42	17	87	52.7 ± 17.6
Clinical Stage II	24	43	81	59.5 ± 11.1
Clinical Stage III	23	29	85	59.7 ± 14.0
Clinical Stage IV	10	34	78	58.1 ± 13.3

Written informed consent was obtained from all patients prior to sample collection. All clinical investigations were conducted in accordance with the Declaration of Helsinki, and the study was approved by the Ethics Committee of the University of P. J. Šafárik in Košice, Medical Faculty (20 N/2016).

## Data Availability

The data presented in this study are available on request from the corresponding authors. The data are not publicly available due to ethical restrictions.
